# Effects of Energy Relaxation via Quantum Coupling Among Three-Dimensional Motion on the Tunneling Current of Graphene Field-Effect Transistors

**DOI:** 10.1186/s11671-015-1039-4

**Published:** 2015-08-12

**Authors:** Ling-Feng Mao, Huansheng Ning, Xijun Li

**Affiliations:** School of Computer and Communication Engineering, University of Science and Technology Beijing, Beijing, 100083 China; Wenzhou Meta Optics Corporate Limited, Wenzhou, 325000 China

**Keywords:** Graphene, Energy relaxation, Tunneling, 85.30.Tv, 85.35.-p, 73.63.-b

## Abstract

We report theoretical study of the effects of energy relaxation on the tunneling current through the oxide layer of a two-dimensional graphene field-effect transistor. In the channel, when three-dimensional electron thermal motion is considered in the Schrödinger equation, the gate leakage current at a given oxide field largely increases with the channel electric field, electron mobility, and energy relaxation time of electrons. Such an increase can be especially significant when the channel electric field is larger than 1 kV/cm. Numerical calculations show that the relative increment of the tunneling current through the gate oxide will decrease with increasing the thickness of oxide layer when the oxide is a few nanometers thick. This highlights that energy relaxation effect needs to be considered in modeling graphene transistors.

## Background

Graphene, a promising carbon-based electronic material, has been emerging as both a unique system for fundamental studies of condensed matter and quantum physics and a fascinating building block for integrated circuits in the age of post-silicon devices. Two-dimensional graphene has a zero band gap and linear energy-momentum dispersion. Graphene’s linear energy − momentum dispersion causes its charge carriers to behave as massless Dirac fermions that can travel at a speed of 10^6^ m s^−1^ [1]. The technical interest stems mostly from the fact that both carrier concentration and type (either electrons or holes) can be controlled by an applied field and that the carriers possess exceptionally high mobility. Graphene exhibits remarkable room temperature mobility in the order of 20,000–200,000 cm^2^ V^−1^ s^−1^ [2] and high carrier mobility even up to 42,000 cm^2^ V^−1^ s^−1^ was also observed [3]. The values range from 10,000–15,000 cm^2^ V^−1^ s^−1^ for exfoliated graphene on SiO_2_ substrate to over 100,000 cm^2^ V^−1^ s^−1^ for suspended samples, making graphene a potential candidate for ultra-fast electronic devices [4].

Hot electron phenomena have become important for understanding all modern semiconductor devices. In hot electron transport, elastic collisions dominant its momentum relaxation, whereas inelastic interaction with phonons determines its energy relaxation. Electro-thermal analysis is used for predicting the heat generation of semiconductor devices. The governing equations of electro-thermal analysis consist of the continuity equation, momentum conservation equation, and energy conservation equation [5]. The non-equilibrium nature of electrons and phonons becomes critical for devices with gate lengths typically shorter than 1 μm [6]. Non-equilibrium state between electron temperature and lattice temperature results that electron temperature becomes much higher than lattice temperature due to electro-thermal effect [5–7]. The absorption of radiation by the electron system also boosts the electron temperature much higher than the lattice temperature, and the temperature equilibration process is governed by the energy relaxation time [8]. The electron temperature determined by experiments rises quickly with the increasing applied electric field. The electron effective mass m^*^ = 0.012 m_0_ (*m*_0_ is the free electron mass) that is magnetic field independent in monolayer graphene has been obtained from the temperature dependent amplitude of SdH oscillation. It has reported that in a graphene device, the energy relaxation time is about 1 ps for carrier-dense samples [9].

The parabolic band effective mass theory is used to investigate the quantum feature of electrons in the inversion layer of metal-oxide-semiconductor field-effect transistors (MOSFETs), quantum well, and superlattices. And one can notice that most studies assume that electron motions in three spatial directions are described by decoupled Schrödinger equations. It has been pointed out that this decoupled motion approximation results in a large error in evaluating the tunneling current and quantization of the inversion layer when the channel electron velocity is higher than the thermal velocity [10, 11]. It has also been verified that such an approximation can result in an error larger than 50 % for the inversion electron density and over 20 % for the gate leakage current [10, 11]. Precisely, modeling tunneling current through the HfO_2_ and SiO_2_ stacks also needs quantum coupling effects correction [12]. However, the relative increase in gate leakage current for HfO_2_/SiO_2_ gate dielectric stacks in experiments is still higher than predictions from motion-coupled quantum model which neglects the energy relaxation of hot electrons [13]. This implies that it is not enough to model the gate leakage current without considering energy relaxation of hot electrons in the device. Note that an electron temperature is well above the lattice temperature, and thus, energy relaxation of the hot electrons can increase the tunneling current through the gate oxide. In this paper, we report how this energy relaxation affects graphene field-effect transistors (FETs).

For graphene FETs, the tunneling current through a 50-nm SiO_2_ layer at 3.5 V, which is the maximal gate-source voltage, is found to be 3 μA/cm^2^ [14]. This gate leakage current is much higher than that of a silicon FET (for a silicon FET, the tunneling current through 4.3-nm oxide layer is less than 0.3 μA/cm^2^ at the same gate bias of 3.5 V [15]). Proceed from our previous studies [16, 17], which do not count energy relaxation between hot electrons and lattice, we will highlight the physical process of energy relaxation of hot electron during tunneling through the gate dielectrics of a graphene transistor.

## Method

The three-dimensional full time independent Schrödinger equation can be written as [18]

1 $$ \left(\frac{1}{2{m}_{\perp }}{\overset{\frown }{p}}_{\perp}^2+\frac{1}{2{m}_z}{\overset{\frown }{p}}_z^2+\varphi \left(x,y,z\right)\right)\psi \left(x,y,z\right)=E\psi \left(x,y,z\right) $$

where *ϕ*(*x*,*y*,*z*) represents the potential energy, *m*_⊥_ and *m*_z_ denote the mass in and perpendicular to the plane of the graphene/gate oxide interface plane at the bottom of the conduction band of graphene, respectively, *z* is the tunneling direction which is perpendicular to the graphene/gate oxide interface, *p*_⊥_ and *p*_z_ represent the electron momentum operators perpendicular to and parallel with the graphene/gate oxide interface, respectively, and *E* is the total energy of an electron. To calculate the tunneling current in a graphene FET, the Schrödinger equation for the electron motion in the *xy* plane keeps unchanged; thus, we need to solve the Schrödinger equation along the *z* direction. At the same time, one can note that *m*_⊥_ and *m*_z_ (in and perpendicular the plane) change along the *z* direction because a graphene FET is a multi-layer structure, which has layer dependent effective electron mass along *z* direction. Therefore, Eq. 1 can be rewritten as

2 $$ \left(\frac{1}{2{m}_{\perp }(z)}{\overset{\frown }{p}}_{\perp}^2+\frac{1}{2{m}_z(z)}{\overset{\frown }{p}}_z^2+\varphi (z)\right)\psi (z)=E\psi (z) $$

where *ϕ*(*z*) represents the potential energy along *z* axis. Note that $\left[{\overset{\frown }{p}}_{\perp },\overset{\frown }{H}\right]=0$ can be obtained from Eq. 2 ($\overset{\frown }{H}$ is the Hamiltonian operator). It denotes that the transverse momentum of the electron (${\hslash}^2{k}_r^2$) conserves in the tunneling process. The wave function for Eq. 2 will be the form $\psi = \exp \left(i\frac{{\overset{\rightharpoonup }{p}}_{xy}}{\hslash}\cdot \overset{\rightharpoonup }{r}\right)\psi (z)$ ($\frac{{\overset{\rightharpoonup }{p}}_{xy}}{\hslash }$ and $\overset{\rightharpoonup }{r}$ are the transverse-wave vector and the displacement vector in the *x*-*y* plane). According to the conservation of the total energy and transverse momentum of a tunneling electron, the longitudinal energy of a tunneling electron in the graphene is ${E}_z^g=E-\frac{{\left({\overset{\rightharpoonup }{p}}_{xy}\right)}^2}{2{m}_{z-g}^{*}}$ and that in the oxide is ${E}_z^{ox}=E-\frac{{\left({\overset{\rightharpoonup }{p}}_{xy}\right)}^2}{2{m}_{ox}^{*}}$ (*E* is the total energy of a tunneling electron). Thus, in the active material (graphene) and the gate oxide (we substitute ${E}_z^g$ for ${E}_z^{ox}$ in the Schrödinger equation across the gate oxide layer), the Schrödinger equation along tunneling direction can be written respectively

3 $$ \left[-\frac{\hslash^2}{2{m}_{z-g}^{*}}\frac{\partial^2}{\partial {z}^2}+\varphi (z)\right]\psi (z)={E}_z^g\psi (z) $$

4 $$ \left[-\frac{\hslash^2}{2{m}_{ox}^{*}}\frac{\partial^2}{\partial {z}^2}+\left(\varphi (z)-\frac{\hslash^2{k}_r^2}{2{m}_{\perp -g}^{*}}\left(1-\frac{m_{\perp -g}^{*}}{m_{ox}^{*}}\right)\right)\right]\psi (z)={E}_z^g\psi (z) $$

where ${m}_{z-g}^{*}$ and ${m}_{\perp -g}^{*}$ are the longitudinal and transverse masses of electron in the graphene, and ${m}_{ox}^{*}$ is the effective electron mass of the gate oxide. The reason why such a 1D Schrödinger equation can be used to characterize the tunneling current of a 3D device is because a tunneling electron must obey the law of the total energy conservation and the transverse momentum conservation. Therefore, the tunneling current calculated from the 1D Schrödinger equation under the conservation conditions has included the effects of the transverse motion of tunneling electrons on the tunneling current. The average energy of electron can be written as $E=\frac{1}{2}{m}^{*}{v}_{drift}^2+\frac{3}{2}{k}_B{T}_{\mathrm{e}}$, where *V*_drift_ is drift velocity, *k*_B_ is Boltzmann constant, and *T*_e_ the electron temperature [19]. Usually the thermal velocity is much higher than the drift velocity, and the average energy can be well approximated by the thermal energy only [19]. Under this assumption, the average electron energy in the plane parallel to the graphene/gate oxide interface can be obtained as $\frac{\hslash^2{k}_r^2}{2{m}_{\perp -g}^{*}}={k}_B{T}_e$. In a FET, a channel electric field cannot only cause channel electrons to drift but also change their energy of disordered thermal motion. Thus, the relation between the electron temperature *T*_e_ and lattice temperature *T*_L_ is [20]

5 $$ {T}_e={T}_L+\frac{2}{3}\frac{q{\tau}_e{\mu}_e}{k_B}{E}_{ch}^2 $$

where *μ*_e_ is the mobility, *τ*_e_ the energy relaxation time, *E*_ch_ the electric field along the channel, and *q* the electron charge. Then the Schrödinger equation in the gate oxide can be rewritten as

6 $$ \left[-\frac{\hslash^2}{2{m}_{ox}^{*}}\frac{\partial^2}{\partial {z}^2}+\varphi (z)-{k}_B\left({T}_L+\frac{2q{\tau}_e{\mu}_e{E}_{ch}^2}{k_B}\right)\left(1-\frac{m_{\perp -g}^{*}}{m_{ox}^{*}}\right)\right]\psi (z)={E}_z^g\psi (z) $$

According to Eq. 6, an effective potential $\varphi (z)-{k}_B\left({T}_L+\frac{2q{\tau}_e{\mu}_e{E}_{ch}^2}{k_B}\right)\left(1-\frac{m_{\perp -g}^{*}}{m_{ox}^{*}}\right)$ other than *φ*(*z*) applied to the tunneling electron in the gate oxide mainly depends on the temperature of channel electrons, energy relaxation rate, electron mobility, and effective electron mass.

For graphene FETs, electron or hole density in the channel induced by the voltage across the gate oxide can be written as [21]

7 $$ {n}_{\mathrm{ch}}=\frac{C_{ox}V}{q}=\frac{\varepsilon_0\varepsilon V}{t_{ox}q} $$

where *C*_ox_ and *t*_ox_ are the capacitance and the thickness of the gate oxide, respectively, *V* is the voltage across the gate oxide layer, *ε*_0_ is vacuum permittivity, and *ε* the dielectric constant of the gate oxide. With setting the bottom of the conduction band of the graphene as zero energy point, the electrons density in the graphene layer of a graphene FET can be obtained as

8 $$ {n}_{2D-G}={\displaystyle \underset{0}{\overset{\infty }{\int }}\frac{E}{\pi {\left(\hslash {v}_0\right)}^2}\frac{1}{1+ \exp \left(\left(E-{E}_F\right)/{k}_BT\right)}dE} $$

At last, an electron current will form because of the imbalance between the Fermi level of the gate and that of substrate induced by the voltage across the gate oxide, and it can be written as [22]

9 $$ I\left(E,V,T\right)=q{\displaystyle \int v(E)T\left(E,V\right)N(E)\left(f\left(E-\left({E}_F+qV\right),T\right)-f\left(E-{E}_F,T\right)\right)}dE $$

where *f* is the Fermi-Dirac distribution function, *N*(*E*) the density of states, *T*(*E*,*V*) the transmission probability, and *v*(*E*) the electron velocity along the tunneling direction.

After obtaining the effective potential barrier of the graphene transistors, we can divide the potential barrier into *N* partial sub-barriers. When a voltage across the gate oxide is applied, the potential barrier shape of the gate oxide will change from square to triangular. Thus, the shape of all sub-barriers is trapezoid as this triangular potential barrier is divided into *N* partial sub-barriers. For trapezoid potential barrier, Airy functions secure an analytical and exact expression of the transmission probability. This is why we use Airy function approach to solve one-dimensional Schrödinger equation to calculate the transmission coefficient of tunneling electrons. Considering the boundary conditions that the wave function and quantum current density are continuous at graphene/gate oxide interface, the transmission probability can be calculated according to the following equation [23, 24]

10 $$ T=\frac{k_R{m}_0^{*}}{m_{N+1}^{*}{k}_L}\frac{1}{\left|{S}_{11}\right|} $$

where *S*_11_ represents the row 1 and column 1 element of the transfer matrix *S* which is

11 $$ \begin{array}{l}S={\left[\begin{array}{cc}\hfill 1\hfill & \hfill 1\hfill \\ {}\hfill \frac{i{k}_L}{m_0^{*}}\hfill & \hfill -\frac{i{k}_L}{m_0^{*}}\hfill \end{array}\right]}^{-1}\left[\begin{array}{cc}\hfill Ai\left({y}_1\left({x}_1\right)\right)\hfill & \hfill Bi\left({y}_1\left({x}_1\right)\right)\hfill \\ {}\hfill \frac{\gamma_1}{m_1^{*}}A{i}^{\hbox{'}}\left({y}_1\left({x}_1\right)\right)\hfill & \hfill \frac{\gamma_1}{m_1^{*}}B{i}^{\hbox{'}}\left({y}_1\left({x}_1\right)\right)\hfill \end{array}\right]\times {\displaystyle \prod_{j=2}^{N-1}{M}_j}\times \\ {}{\left[\begin{array}{cc}\hfill Ai\left({y}_N\left({x}_N\right)\right)\hfill & \hfill Bi\left({y}_N\left({x}_N\right)\right)\hfill \\ {}\hfill \frac{\gamma_N}{m_N^{*}}A{i}^{\hbox{'}}\left({y}_N\left({x}_N\right)\right)\hfill & \hfill \frac{\gamma_N}{m_N^{*}}B{i}^{\hbox{'}}\left({y}_N\left({x}_N\right)\right)\hfill \end{array}\right]}^{-1}\left[\begin{array}{cc}\hfill 1\hfill & \hfill 1\hfill \\ {}\hfill \frac{i{k}_R}{m_{N+1}^{*}}\hfill & \hfill -\frac{i{k}_R}{m_{N+1}^{*}}\hfill \end{array}\right]\end{array} $$

where

12 $$ \begin{array}{l}{M}_j={\left[\begin{array}{cc}\hfill Ai\left({y}_j\left({x}_{j+1}\right)\right)\hfill & \hfill Bi\left({y}_j\left({x}_{j+1}\right)\right)\hfill \\ {}\hfill \frac{\gamma_j}{m_j^{*}}A{i}^{\hbox{'}}\left({y}_j\left({x}_{j+1}\right)\right)\hfill & \hfill -\frac{\gamma_j}{m_j^{*}}B{i}^{\hbox{'}}\left({y}_j\left({x}_{j+1}\right)\right)\hfill \end{array}\right]}^{-1}\times \\ {}\left[\begin{array}{cc}\hfill Ai\left({y}_{j+1}\left({x}_{j+1}\right)\right)\hfill & \hfill Bi\left({y}_{j+1}\left({x}_{j+1}\right)\right)\hfill \\ {}\hfill \frac{\gamma_{j+1}}{m_{j+1}^{*}}A{i}^{\hbox{'}}\left({y}_{j+1}\left({x}_{j+1}\right)\right)\hfill & \hfill \frac{\gamma_{j+1}}{m_{j+1}^{*}}B{i}^{\hbox{'}}\left({y}_{j+1}\left({x}_{j+1}\right)\right)\hfill \end{array}\right]\end{array} $$

13 $$ {y}_j(x)={\left(\frac{2m}{\hslash^2}{F}_j\right)}^{1/3}\left(\frac{V\left({x}_j\right)+{F}_j{x}_j-E}{F_j}-x\right) $$

14 $$ {F}_j=-\frac{V\left({x}_{j+1}\right)-V\left({x}_j\right)}{x_{j+1}-{x}_j} $$

15 $$ {\gamma}_j=\frac{d{y}_j}{dx}={\left(\frac{2{m}_j^{*}}{\hslash^2}{F}_j\right)}^{1/3} $$

where *M*_*j*_ is a (2 × 2) product matrix, *m*_j_^*^ the effective electron mass for *j*th trapezoid barrier, *F*_*j*_ the electric field across the *j*th trapezoid barrier, *V*(*x*_j_) the voltage at the position of *x*_j_, and *Ai* (Ai^’^) and *Bi* (Bi^’^) are Airy functions and their corresponding derivatives, respectively.

The abovementioned method has been applied to study high-energy (hot) electron distribution in the graphene layer induced by its linear energy-momentum dispersion and the gate leakage current in graphene FETs [25]. Firstly, we calculate the electron temperature increment in the graphene layer caused by the energy relaxation process. Then the impacts of the quantum coupling among channel electron motions in three dimensions have been quantified. Linear energy-momentum dispersion of graphene electrons has been taken in all calculations of tunneling current in this article.

## Results and Discussion

In this work, dielectric constant of 2.4 has been used in graphene field-effect transistor [26]. The thickness of 0.34 nm is used for a single atomic layer of graphene [27]. Used as the work function for graphene were 4.5 eV according to [28] and 4.4 eV according to [29]. The gate oxide SiO_2_ has an electron affinity of 0.9 eV and relative dielectric constant of 3.9. The barrier height used in this study is 3.5 eV. Electron mass of 0.012 *m*_0_ in 2D graphene according to [30], and 0.5 *m*_0_ in the gate oxide, was used in all calculations. Because graphene is a 2D crystal, there is no band-structure in the tunneling direction which is perpendicular to the graphene plane. This is the reason why we use free electron mass along such a direction in all calculations. The energy relaxation time of 1 ps [9] and the mobility of the order of 20,000–200,000 cm^2^ V^−1^ s^−1^ [2–4] are used in the calculations. International units have been used in all calculations.

**Fig. 1:**
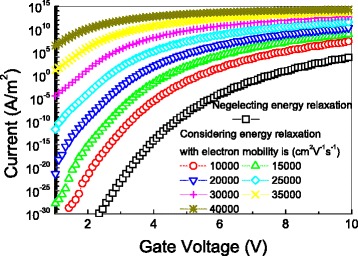
The tunneling current as a function of the gate voltage with the oxide thickness of 10 nm. The channel electric field is 10 kV/cm, the energy relaxation time is 1 ps, and the device temperature is 300 K

How the tunneling current through the gate oxide with and without considering the energy relaxation of channel electrons changes with the gate voltage at different electron mobility is illustrated in Fig. 1. Figure 1 clearly shows that the energy relaxation of channel electrons can lead to huge enhancement (over three orders) in the tunneling current through the gate oxide of two-dimensional graphene FETs with different electron mobility. This figure also shows that the electron mobility can induce a large increase in the gate leakage current. It should be pointed out that tunneling current of devices with larger electron mobility can be more seriously affected by energy relaxation.

**Fig. 2:**
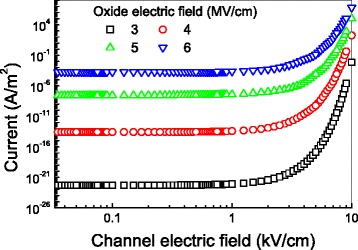
The tunneling current as a function of the channel electric field with the oxide thickness of 10 nm. The electronic mobility is 20,000 cm^2^ V^−1^ s^−1^, the oxide field is 5 MV/cm, the energy relaxation time is 1 ps, and the device temperature is 300 K

Figure 2 depicts the tunneling current through the gate oxide as a function of the channel electric field. In the figure, the tunneling current exponentially increases with channel electric field when the channel electric field is higher than 1 kV/cm. This figure also clearly shows that to maintain a stable and high performance, a graphene FET is better to operate with low gate field (3 to 4 MV/cm) and a low channel electric field of 1 kV/cm.

**Fig. 3:**
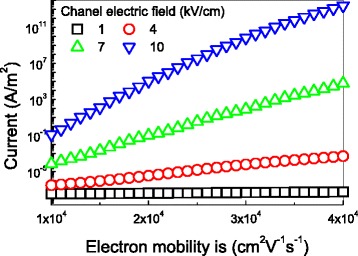
The tunneling current as a function of the electron mobility with the oxide thickness of 10 nm. The oxide field is 5 MV/cm, the electronic mobility is 20,000 cm^2^ V^−1^ s^−1^, the energy relaxation time is 1 ps, and the device temperature is 300 K

The effects of the electron mobility on the gate leakage current of a graphene field-effect transistor are given in Fig. 3. It can be found that the tunneling current exponentially increase with the electron mobility when the channel electric field is larger than 1 kV/cm. This implies that energy relaxation must be considered for large channel electric field. It is very critical for a high frequency graphene FET (high electron mobility) to work at a channel field lower than 1 kV/cm to remain a low noise status.

**Fig. 4:**
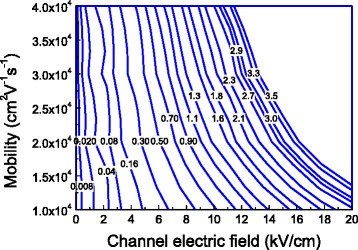
The contour of the reduction in the barrier height in the unit of eV caused by the electron mobility and channel electric field due to the energy relaxation of electrons when the energy relaxation time is 1 ps

The reduction in the barrier height caused by the channel electric field and the electron mobility was detailed shown in Fig. 4. Larger reduction in the barrier height results in a higher tunneling current. This is consistent with the conclusion from Figs. 2 and 3. Figure 4 explains that the tunneling current becomes larger when the channel electric field is larger than 1 kV/cm because the reduction in the barrier height caused by energy relaxation of channel electrons is large enough to affect the tunneling current.

**Fig. 5:**
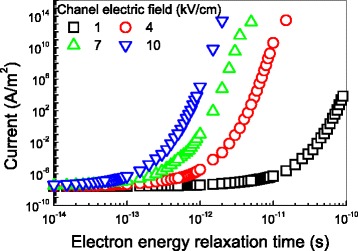
The tunneling current as a function of the energy relaxation time of electron with the oxide thickness of 10 nm. The oxide field is 5 MV/cm, the electronic mobility is 20,000 cm^2^ V^−1^ s^−1^, the channel field is 5 kV/cm, and the device temperature is 300 K

The electron energy relaxation time impacting on the tunneling current through the gate oxide at different channel electric fields is illustrated in Fig. 5. This figure clearly demonstrates that the tunneling current keeps negligible at even high channel field up to 10 kV/cm when the energy relaxation time is shorter than 0.1 ps. It implies that the effects of energy relaxation on the barrier height can be neglected when the energy relaxation time is shorter than 0.1 ps. However, for devices with the relaxation time longer than 1 ps and the channel electric field less than 1 kV/cm, energy relaxation is needed to be considered for ensuring high device performance.

**Fig. 6:**
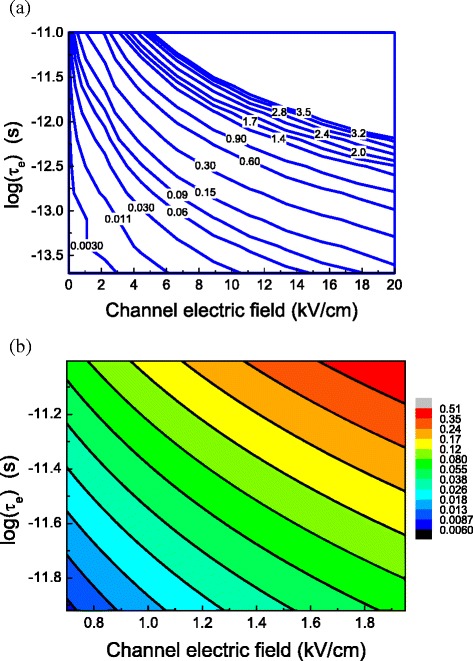
**a** The contour of the reduction in the barrier height in the unit of eV caused by the energy relaxation time and channel electric field due to the energy relaxation of electrons when the electronic mobility is 20,000 cm^2^ V^−1^ s^−1^, **b** an enlarge figure of the reduction in the barrier height range around 1 kV/cm and 1 ps

The reduction in the barrier height as a function of the electron energy relaxation time at different channel electric fields is depicted in Fig. 6a. This figure clearly shows that the reduction in the barrier will increase with the energy relaxation time at a given channel electric field. And it also shows that such a reduction in the barrier height can be neglected for energy relaxation time shorter than 0.1 ps and for channel electric field lower than 1 kV/cm. Figure 6b gives the detail of the reduction in the barrier height when the electron energy relaxation time is over 1 ps and the channel electric field is around 1 kV/cm. This figure clearly shows a little reduction in the barrier height when the electron energy relaxation time is less than 1 ps and the channel electric field is less than 1 kV/cm.

**Fig. 7:**
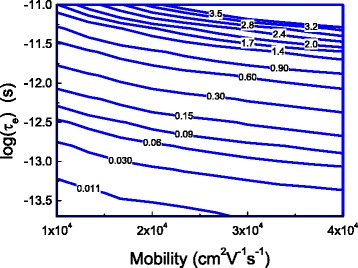
The contour of the reduction in the barrier height in the unit of eV caused by the energy relaxation time and channel electron mobility due to the energy relaxation of electrons when the channel electric field is 5 kV/cm

Figure 7 depicts the effects of a combination of the energy relaxation time and electron mobility on the barrier height at a given channel electric field. This figure clearly shows that energy relaxation effect must be taken into consideration in any device with either its energy relaxation time and/or its electron mobility is large enough.

Figures 3, 6, and 7 give quantitatively explain for which devices the effects of the electron energy relaxation on the tunneling current can be neglected. This is because in these devices, either their channel electrical field is lower than 1 kV/cm or their electron energy relaxation time is shorter than 0.1 ps. They also outline the optimized parameters of future graphene devices.

**Fig. 8:**
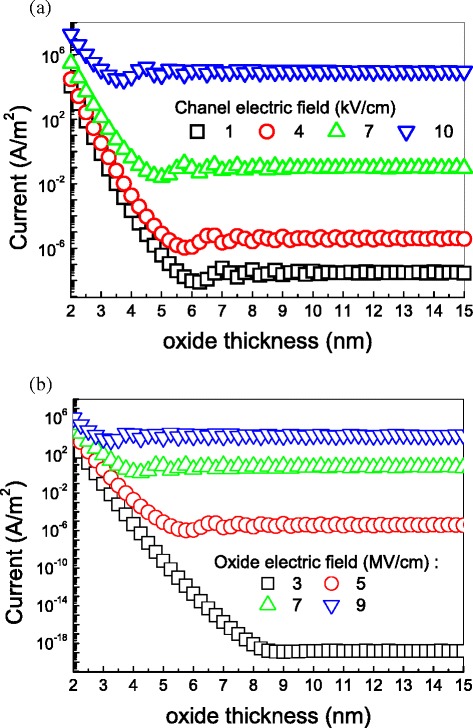
**a** Tunneling current as a function of the gate thickness at different channel fields. The gate field is 5 MV/cm, the electronic mobility is 20,000 cm^2^ V^−1^ s^−1^, the energy relaxation time is 1 ps, and the device temperature is 300 K. **b** Tunneling current as a function of the gate thickness at different oxide fields. The channel electric field is 4 kV/cm

Figure 8a demonstrates how the tunneling current through the gate oxide changes with the decreasing oxide thickness. This figure illustrates that the tunneling current will increase largely with decreasing oxide thickness when the oxide is less than 6 nm. For a gate oxide thicker than 6 nm, further increase in the gate oxide thickness helps little to reduce tunneling current of a graphene FET. This is also important for device fabrication. We point out that a 6-nm gate oxide is the optimal for graphene FETs. As a comparison, the tunneling current as a function of the oxide thickness at different electric field across the gate oxide layer is given in Fig. 8b. Similar conclusion to that from Fig. 8a can be obtained.

It is well known that metal contacts (both source/drain and gate metal) are crucial in the creation of high-performance graphene FETs [31]. The impact of electrodes on the performance of graphene transistor (for example the metal-induced doping of graphene) is needed to be clarified [32]. Such a topic is very important, and it is also worth studying how metal contacts (both source/drain and gate metal) affect on the carrier transport in the channel (including its impacts on the energy relaxation of channel electrons or electro-thermal effects in graphene transistors). Such a topic is our future study project, and relative study may be reported later. But this paper only deals with the case of the energy relaxation of channel electron impacts on the tunneling current through the gate oxide layer. Thus, we do not discuss the impacts of metal on the performance of graphene FETs.

## Conclusions

In conclusion, energy relaxation effect on the tunneling current through the gate oxide of a graphene field-effect transistor has been theoretically investigated. The quantum coupling among the three electron spatial motion directions can result in a larger reduction in the barrier height. Note that the energy relaxation of electrons may largely increase electron temperature. The energy relaxation due to electron thermal motion along the graphene channel will lower the effective barrier height which enhances the gate leakage current. This study points out that the increase in the electron mobility, channel electric field, and the energy relaxation time always leads to higher leakage current in a graphene field-effect transistor. Based on quantified results, this paper also details how to secure high-performance graphene device and ICs. Devices with energy relaxation of time shorter than 0.1 ps or a working channel field of less than 1 kV/cm must be secured. It is also worth studying how electro-thermal effects affect the performance of multi-stack gated 2X nano-meter or 1X nano-meter MOSFETs and relative study will be reported later.

## Abbreviations

FET: field-effect transistor
1D: one dimensional
3D: three dimensional
